# Treatment decision-making and quality of life versus length of life preferences of older patients with early stage cancer: A systematic review

**DOI:** 10.1016/j.jgo.2025.102773

**Published:** 2025-11

**Authors:** Charlene Martin, Jessica Banks, Nicolò Matteo Luca Battisti, Hilary Bekker, Adrian Edwards, Lynda Wyld, Jenna Morgan

**Affiliations:** a Division of Clinical Medicine, School of Medicine and Population Health, The University of Sheffield, UK; b Department of Medicine, The Royal Marsden NHS Foundation Trust, London, UK; c Leeds Institute of Health Sciences, School of Medicine, University of Leeds, UK; d Division of Population Medicine, School of Medicine, Cardiff University, Neuadd Meirionnydd, Heath Park, Cardiff CF14 4YS, UK

**Keywords:** Decision-making, Quality of life, Geriatric oncology

## Abstract

**Introduction:**

To fully consider the preferences and information needs of older adults, cancer treatment decision-making discussions should take a patient-centred approach. Some older patients may place more value on maintaining quality of life over the continuation of life-prolonging treatments, even when the cancer is early-stage and potentially curable. Decision support tools can play a role in facilitating discussions around treatment trade-offs. The objective of this review is to examine the literature on the treatment decision-making preferences of patients aged 70 and older with early-stage, potentially curable, cancer.

**Materials and Methods:**

MEDLINE OVID, CINAHAL, APA PsycINFO, Scopus, and Cochrane databases were systematically searched in January 2025. Published literature focusing on quality and length-of-life decision-making, and the use of decision support tools aimed towards older adults diagnosed with early-stage cancer, were included. Two authors performed full-text selection and quality appraisal. Data were synthesized according to themes, using the Framework Approach.

**Results:**

From 1476 screened records, a total of 14 studies were included. Five key themes were identified: Information needs; Treatment preferences; Trade-offs (treatments, quality and length-of-life); Decision-making involvement; Available decision support interventions.

**Discussion:**

Evidence suggests that older patients would benefit from receiving information about both quality and length-of-life when making cancer treatment decisions. Quality of life concerns including physical wellbeing, autonomy, and symptom burden were factors considered by patients. Decision support tools have the potential to assist in trade-off discussions, however, few have been developed to balance trade-offs between quality and length-of-life.

**Registration:**

PROSPERO: CRD42025626454.

## 1 Introduction

Older age is one of the most potent risk factors for cancer [1], with older adults constituting the largest proportion of patients with cancer worldwide [2]. Patients with frailty, comorbidity, and cancer experience fewer benefits and more complications from cancer treatments, irrespective of their cancer diagnosis and the therapy options available. Ageing can influence cancer progression, adding complexity to the management of older patients who are nearer to the end of their natural lifespan [3]. The presence of comorbidities and diminished functional reserves can also affect treatment tolerance in older patients [4], particularly those diagnosed in later life.

Cancer treatments carry the risk of severe side effects, and while they may extend life, they can also impede the ability to maintain a good quality of life (QoL) [5]. Whilst many cancer treatment side effects are transient (nausea, fatigue due to chemotherapy, pain due to surgery), some can be long-term. These may include peripheral neuropathy from chemotherapy, lymphoedema from axillary surgery, and stomas from bowel surgery, all of which can have lasting QoL and functional implications. These treatment impacts are especially pertinent for older adults, as not all return to their baseline QoL or functional status after completing cancer treatments [6,7]. The gradual loss of independence both during and following cancer treatment is well-documented [7,8], and is therefore much more likely to have enduring effects on older, frailer patients in the long-term [7]. Cancer treatments, such as chemotherapy and hormone therapies, can also have an impact on cognitive function [9,10], which is a major fear for many older adults. Cognitive decline is often perceived as a threat to QoL, particularly independence and autonomy [11]. In one cohort study, 72% of older adults with cancer rated cognition preservation as a high priority [12] while other studies have reported that many older patients ranked cognitive function above survival [13,14].

Given these concerns, particularly around maintaining function and autonomy, QoL becomes a central consideration in treatment decision-making (DM) for many older patients. In two previous reviews, QoL considerations were strongest amongst older patients [15], with Segher and colleagues [16] reporting that in patients ≥70 years, QoL was ranked more highly than survival; however both of these reviews included a large proportion of papers in the palliative setting where treatment outcomes are different from the curative setting. Preserving QoL can pose a substantial challenge for healthcare professionals (HCPs), especially where some oncological treatments carry a high risk of toxicity, and age-specific data on how treatments affect QoL are unavailable [17]. Older adults with cancer may also be prone to significant risk of under- and overtreatment [18]. One common obstacle is the lack of evidence available to guide personalized cancer care in older patients, meaning that some clinicians may choose to deviate from standard treatment. Contributing factors for this include limited data on treatment outcomes in older populations, few evidence-based guidelines, and generalized/younger age biased treatment approaches.

Recognising the heterogeneity amongst patients, new frameworks and standards for decision support tools have been developed to help patients consider their options [19,20]. Decision support resources [21] can help HCPs to provide tailored information that caters to the individual needs and preferences of patients with various cancer types [22,23]. There is also increasing evidence that decision support tools can improve patient knowledge of, and confidence in, treatments [[24], [25], [26]], as well as support patient engagement with oncology consultations [27]. Although few decision aids have been developed based on data from older populations [28], this is improving [29].

Despite increasing attention to this topic, there is a scarcity of literature addressing QoL trade-offs in older adults with cancer, with older populations in particular being historically underrepresented in clinical research [30]. Previous research has highlighted a clear gap in the literature concerning nuanced age-specific studies that define the QoL drivers of older patients [31] and QoL trade-off understandings [15,32]. As demographics trend towards an ageing population, there is a growing need to understand the treatment DM preferences of older adults and what QoL means to different groups of patients. In doing so, HCPs can encourage a patient-centred approach, assisting patients to make treatment decisions that align with their personal preferences and beliefs.

This review aims to:

1. Determine the quality and length-of-life priorities with respect to treatment DM amongst older patients who have been diagnosed with early, potentially curable (i.e., non-metastatic) cancer.
2. Clarify what older adults with early cancer understand by the term QoL and whether the meaning differs between patient groups.
3. Identify cancer decision support tools aimed at helping older patients make quality and length-of-life treatment decisions.

This review was undertaken as part of the groundwork for a study that aims to develop a new tool to help doctors better understand whether older patients with early-stage, potentially curable, cancer prioritize a longer life or maintaining their QoL when making cancer treatment decisions.

## 2 Methods

### 2.1 Search strategy

A comprehensive literature search was conducted using five databases (MEDLINE OVID, CINHAL, APA PsycINFO, Scopus, and Cochrane) from inception to January 2025. There were no date limits set. A preliminary search was carried out to identify keywords and develop the search strategy. A librarian assisted the development of the search strategy (Supplemental Data 1). Citation lists of screened papers were searched with forward citation tracking to identify additional studies. Papers were screened using Rayyan software [33].

### 2.2 Selection process

Titles and abstracts were reviewed independently by two researchers (CM and JB) using the SPIDER Tool [34] (Table 1). The inclusion and exclusion criteria are displayed in Table 2. Articles with at least two reviewer votes were reviewed in full text. Where the inclusion decision was in conflict, a third reviewer (JM) made the final decision to review the full text. Full-text articles were reviewed collaboratively by two reviewers (CM and JM). Where it was unclear if a study met the inclusion criteria, attempts were made to contact the author team via email to clarify. Author teams were only contacted once.

**Table 1:** SPIDER tool.

SPIDER	Elements of SPIDER applied to search strategy
S – Sample	Older patients with cancer
PI – Phenomenon of interest	Quality versus length of life information preferences
D – Design	Published literature of any research design, grey literature forward citation searching
E – Evaluation	Preferences for outcome format; influences in decision-making; trade-offs; decision support tools that support decision-making
R – Research Type	Qualitative and quantitative studies; mixed method studies

**Table 2:** Inclusion and exclusion criteria.

Inclusion criteria	Exclusion criteria
• Qualitative, quantitative, mixed methods • Adults > age 18 • Older patients (e.g., over 65 years or 70 and above) **must** be a part of the study population: either the majority (>50%) of the sample, form a separate comparative group, or the mean/median age of the study population should be ≥65 years. • Focusing on length of life versus quality of life in patients with cancer • Any paper that refers to decision-making in a cancer setting • Any study that uses a decision aid or tool as part of their intervention • Any study that aids shared decision making • English language • Any gender • Any cancer type	• Conference abstracts • Protocols • Metastatic patient population **should not** be in the majority (population majority should be early stage/curable cancer) • Paper unavailable in English Language

### 2.3 Quality and equity, diversity, and inclusion assessment

Two reviewers (JM and CM) collaboratively assessed the methodological quality of studies using the Mixed Methods Appraisal Tool (MMAT) [35] and undertook an evaluation of equity, diversity, and inclusion (EDI) across the included studies using the PROGRESS-Plus assessment [36].

### 2.4 Synthesis methods

Data were synthesized according to themes using NVivo software (version 1.7.1). This process was guided by the Framework Approach [28,[37], [38]]. Data synthesis was achieved through familiarisation with the data; generation of initial codes; and the refinement of codes into themes. Findings are presented thematically to address the primary research question. The Synthesis without Meta Analysis (SWiM) guideline [39] was followed to critically appraise the data.

Key data were amalgamated into figures where possible for each of the main aims in Microsoft office.

## 3 Results

### 3.1 Study selection

**Fig. 1:**
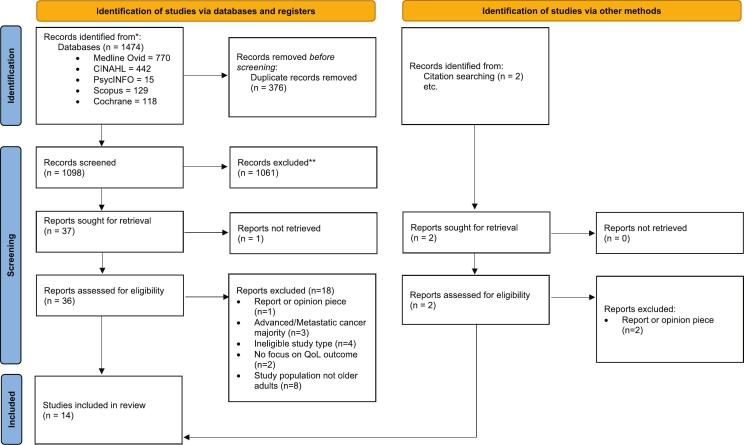
PRIMSA flow-chart. *Consider, if feasible to do so, reporting the number of records identified from each database or register searched (rather than the total number across all databases/registers). **If automation tools were used, indicate how many records were excluded by a human and how many were excluded by automation tools. *From: Page MJ, McKenzie JE, Bossuyt PM, Boutron I, Hoffmann TC, Mulrow CD, et al. The PRISMA 2020 statement: an updated guideline for reporting systematic reviews. BMJ 2021;372:n71. doi:*https://doi.org/10.1136/bmj.n71. For more information, visit: http://www.prisma-statement.org/.

The results of the search are shown in Fig. 1 (PRISMA flow diagram). The initial database search yielded 1474 results, with a further two articles identified via citation searching. Full-text articles (*n* = 38) were reviewed collaboratively by two reviewers (CM and JM), and 25 studies were excluded. Reasons for exclusion are shown in Fig. 1.

Studies were of generally adequate quality, with no studies excluded on the basis of poor quality. The MMAT quality assessment can be seen in Supplemental Table 1.

### 3.2 Study characteristics

A total of 14 articles were included (Table 3). Of these, nine were conducted in Europe [[40], [41], [42], [43], [44], [45], [46], [47], [48]], three in the USA [14,49,50], one in Australia [51], and one study recruited worldwide across a number of regions [52]. Studies were conducted using a quantitative approach (*n* = 9) [14,[43], [44], [45], [46], [47],[49], [50], [51]], surveys (*n* = 6) [14,[43], [44], [45],^49^,^51^], vignettes (*n* = 1) [50], ordinal task ranking (n = 1) [46], and discrete choice experiments (n = 1) [47]. Three qualitative papers [40,42,52] conducted interviews. Other study types included one randomized controlled trial [48], and one mixed method study [41].

**Table 3:** Characteristics of studies (*n* = 14).

Author, date (ref). Location.	*n*	Aim/DSI	Method	Age (years)	Cancer type	Stage
Andersen et al., 2009 [49]. USA.	636	Patient involvement in DM	Quantitative. Cross-sectional survey design.	55 (mean) Assesses age as a factor	Breast	I-IV
Chouliara et al., 2004 [40]. UK	6	Patient perceptions of information and DM.	Qualitative. Semi-structured interviews	65–96 (range) Only older patients	Not reported	Not reported
Dhakal et al., 2022 [14]. USA	100	Cancer treatment preferences of adults with cancer	Quantitative. Survey.	23–89 (range) Specifically presents data for older patients >60 vs younger	Breast, lung, GI, genitourinary, haematological, *others*	66% of the older age group were having treatment with curative intent
Harder et al., 2013 [41]. UK.	58	Older patient experiences and preferences towards information giving and chemotherapy decisions	Mixed Method. Survey and interviews.	70–83 (range) Only older patients	Breast	I-III
Husain et al., 2008 [42]. UK.	21	Older patient attitudes towards PET and surgery	Qualitative. Interviews.	76–91 (range) Only older patients	Breast	I-II
Jansen et al., 2004 [43]. Netherlands.	448	Perceptions of treatment choices	Quantitative descriptive. Survey.	32–89 (range)	Breast	“Early stage” (I-II)
Jorgensen et al., 2013 [51]. Australia.	68	Barriers to chemotherapy use in older patients and preferences for information and DM involvement	Quantitative descriptive. Survey.	25–82 (range) Presents results by age group. Mean age of older group 73.9	Colorectal	Dukes Stages A-D Older age group: 14% Dukes A; 40% Dukes B; 31% Dukes C; 6% Dukes D
Kool et al., 2016 [44]. Netherlands.	350	Whether clinical or patient reported outcomes are most important for QoL	Quantitative descriptive. Survey.	59.34 (mean) Presents analysis of results by age group	Breast	Stage I-III
Noordman et al., 2018 [45]. Netherlands.	100	Investigated factors that influenced patient preference and trade-off in the choice between surgery and active surveillance	Quantitative descriptive. Survey.	61–72 (range) Older patients only.	Oesophageal	II-III
van Tol-Geerdink et al., 2006 [46]. Netherlands.	119	Examine if patients chose the more aggressive of two radiotherapeutic options and what the determinants of the choice are	Quantitative descriptive.	51–84 (range) 70 (mean) Compares different age ranges (>70 vs <70 years)	Prostate	I-II
Watson et al., 2020 [47]. UK.	650	To evaluate and quantify the trade-offs patients make between active surveillance and definitive therapy	Quantitative descriptive.	67 (mean)	Prostate	I-II
Wörns et al., 2024 [52]. USA, France, Germany and Japan.	62 (50 patients; 12 healthcare providers)	To explore the experience of hepatocellular carcinoma in relation to treatment options, DM and goals	Qualitative. Semi-structured interviews.	65 (median)	Hepatocellular carcinoma	Stage A-C
Wyld et al., 2021 [48]. UK.	1339	Evaluated the impact of DESIs for older women with BC. To ascertain if DESIs influenced QoL, survival, decision quality and treatment choice. Age Gap Decision Tool	Randomized controlled trial.	78 (mean)	Breast	I-III
Yellen et al., 1994 [50]. USA.	244	Explored whether treatment preferences of older adults for aggressive cancer therapy differed from younger patients, and if older patients were more or less likely to agree to treatments with high toxicity than their younger counterparts	Quantitative descriptive.	50.7 (mean) Compares older (>65) and younger (<65) patients	Breast, gastrointestinal, lung, lymphoma, other	I-IV

All studies had older patients in their inclusion criteria, however, not all reported an upper age range [44,52]. Of the 14 studies, 36% (*n* = 5) carried out a sub-analysis of outcomes in older patients; however, only one study reported the exact number of participants aged ≥70, with others grouping older patients more broadly (e.g., ≥60). Several papers did not report the cancer type [40] and staging [40] of study participants. Our EDI assessment of studies found variability in participant diversity; only three studies adequately reported at least three PROGRESS-Plus domains, with the most frequently reported being age, gender, and education (Supplemental Table 2).

### 3.3 Results of syntheses

Five key themes were identified:

• **Information needs**
  Older patients have different information needs and patients require information tailored to their individual treatment goals.
• **Treatment preferences**
  Older age may impact treatment decision making, especially for those who prioritize maintaining QoL, preferring less aggressive treatment options.
• **Trade-offs (treatments, QoL, and length-of-life)**
  Older patients tended to place greater emphasis on maintenance of QoL and less on being cured of cancer or prolonging life.
• **Decision-making involvement**
  Older patients' preferred degree of involvement in decision-making varied, but decision-support interventions enhanced shared decision making and impacted on the treatments chosen.
• **Available decision support interventions**
  Our search identified only one decision aid tailored for older patients in the curative cancer setting.

#### 3.3.1 Information needs

Three studies explored patient information needs in respect to treatment outcomes and long/short-term impacts on QoL and length-of-life [41,51,52]. Studies recruited patients with colorectal, breast, and hepatocellular carcinoma.

Wörns and colleagues [52] emphasized the importance of discussing cancer treatment risks and benefits with patients to ensure that outcomes are fully aligned with individual goals. In respect to outcome information, patients desired information on the long-term impact of treatment on QoL [41,51,52], survival benefit [41,51,52], and treatment effectiveness [52]. This was seen most prominently in the study by Jorgensen and colleagues [51] where 60% of older patients ≥65 expressed a preference for detailed information on chemotherapy treatments, which was less than younger patients but not significantly different (76%, *p* = 0.17).

With regards to content needs, patients valued comprehensive information with fewer technical terms, alongside having more time with their care team for discussion [52]. In one study examining factors influencing chemotherapy decisions, older patients generally opted for less information than younger patients, with 50% of older patients (≥65) preferring to receive “as much information as possible” compared 76% of younger patients (≤65) (*p* = 0.03) [51]. In Harder and colleagues' study [41], 80% of patients (aged ≥70) were satisfied with the information received, but some felt there was unclear information on QoL impacts, including independence, cognition, and fatigue. All three studies highlighted the importance of individualized treatment information [41,51,52]. To achieve this, information should be age-sensitive, with information needs assessed regularly [51].

#### 3.3.2 Treatment preferences

Eight studies explored treatment preferences [14,40,42,[45], [46], [47],^50^,^51^] in patients with breast, oesophageal, prostate, lung, gastrointestinal, genitourinary, and haematological cancers.

Treatment preferences were underscored by numerous factors, predominantly age-related considerations [14,40,46,51] and QoL priorities [14,40,42,46,51]. In the study by Dhakal and colleagues [14], older (≥60) patients prioritized minimizing treatment burden, avoiding side-effects, and maintaining current QoL more than younger (≤60) patients, with the over 60 group significantly preferring oral chemotherapy versus IV (*p* = 0.003) and shorter hospital stays (p = 0.03). Similarly, in the study by van Tol-Geerdink and colleagues [46], older age was significantly associated with less aggressive treatments, with 86% of older adults with prostate cancer choosing a lower radiation dose to avoid severe gastrointestinal or genitourinary problems, despite a predicted loss of life expectancy of one year compared to 59% younger patients whose predicted loss of life expectancy was up tofour years (p = ≤001). Although patient age at the time of DM was an important factor for some older patients [51], the women in Husain and colleagues' study recognized that while age *might* have the potential to influence breast cancer DM, this was not a factor for them [42]. Yellen and colleagues [50] also found that age did not significantly influence treatment DM.

Patients were generally more satisfied with their chosen treatment if the side-effects resulted in fewer disturbances to their QoL [42]. *“Carrying on as before”* and *“avoiding disruption in everyday life”* were high priorities for older patients who wished to maintain their QoL [40,42,51], although some were willing to tolerate side effects if this resulted in recovery [40]. Long-term impacts on QoL and performance of activities of daily living (ADL) were important considerations for patients [14], with some preferring a quicker return to normality rather than the pursuit of further treatments [41,42]. Four studies explored the attributes of treatment prioritized most by patients undergoing chemotherapy [14,41,51]. High priority concerns included treatment side-effects [41], fear of death [51], and long-term memory and cognitive impairment [14].

#### 3.3.3 Trade-offs (quality and length-of-life)

Ten studies explored treatment trade-offs [14,40,42,[44], [45], [46], [47],[50], [51], [52]], with four of these examining the trade-off between quality and length-of-life [14,[45], [46], [47]]. Studies recruited patients with prostate, breast, oesophageal, colorectal, and hepatocellular carcinoma.

**Fig. 2:**
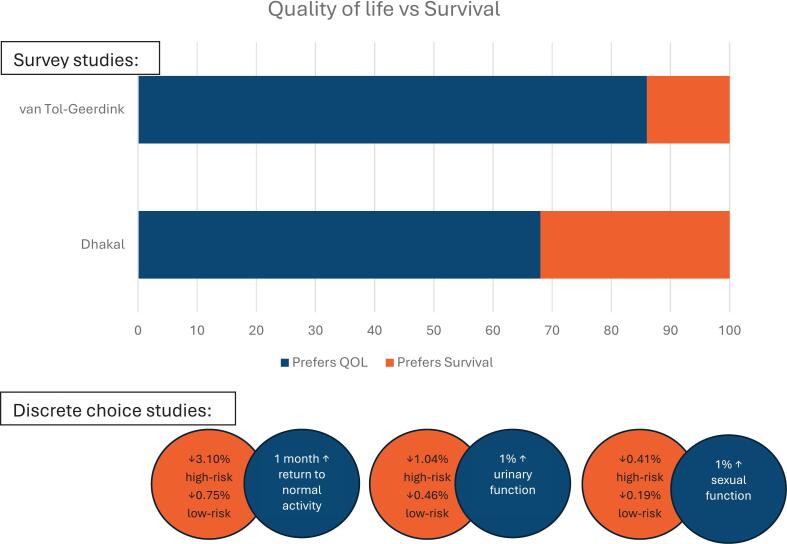
Importance of quality of life vs survival.

Three of the four studies focusing on quality versus length-of-life trade-offs reported an overall priority towards QoL [14,[45], [46], [47]] (Fig. 2). Three QoL domains (physical wellbeing, autonomy, and symptom burden) consistently emerged as patient priorities. Maintaining current level of physical functioning was highlighted in three studies [14,46,47]. Dhakal and colleagues [14] reported that 76% of patients aged ≥60 years prioritized functional well-being and QoL over survival gains, agreeing with the statement, “I would rather live a shorter life than permanently lose my ability to do daily activities such as grooming, eating or self-care.” Symptom burden was highlighted across all four studies [14,[45], [46], [47]], with patients keen to maintain their daily lives without fear of disruptive side effects and discomfort. Older patients placed less importance on sexual function, whereas younger patients considered this a higher priority [14,46]. Whilst older patients in the study by Dhakal and colleagues [14] rated the preservation of cognitive function highly, this was either not assessed or explicitly discussed across the other studies.

In studies comparing young versus older patients, the latter placed greater emphasis on maintenance of QoL [51] and less on being cured of cancer or prolonging life [14]. Older patients were also less likely to prioritize longer disease-free survival and recurrence rates [44] and less likely to choose more aggressive treatments than younger patients [50].

While patients in a handful of studies were willing to undergo effective treatments to extend life at the cost of compromising QoL, these decisions were contingent on a number of factors such as their clinician's advice, the benefit of treatment, and survival gains. In the study by Dhakal and colleagues, a survival benefit of over six months was viewed as a worthwhile trade-off [14]. To avoid the risk of oesophagectomy and improve long-standing impacts on health related-QoL, patients with oesophageal cancer in Noordman and colleagues' study were willing to trade-off a 16% five-year overall survival improvement [45]. Similarly, patients with prostate cancer were willing to accept reductions in survival (up to 3.10% reduction in five-year survival) for improvement in QoL (representing a one-month reduction in time to return to normal activities or 1% improvements in urinary or sexual function) [47]. Kool and colleagues [44] also found that older patients prioritized the avoidance of severe breast symptoms (continuous pain, even with painkillers) with an overall relative importance of 23.22 (95% CI 22.32–24.12) over two-year longer disease-free survival (reducing predicted survival from 11 to 9 years); overall relative importance of 18.30 (95% CI 17.38–19.22).

**Fig. 3:**
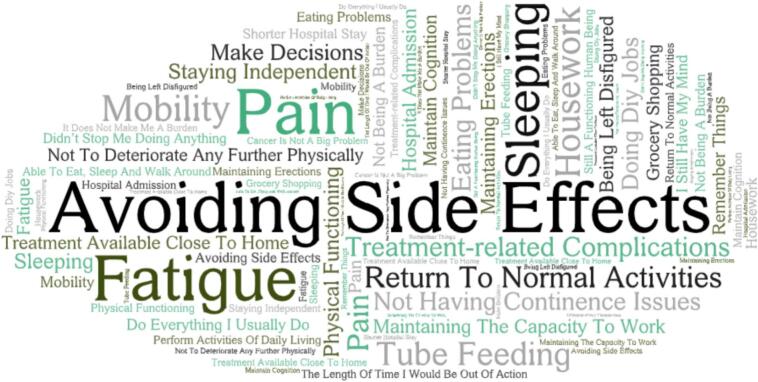
Word cloud depicting the items representing quality of life to older adults with early cancer. Statements relating to the meaning of quality of life were extracted from each paper and similar themes were combined. These themes and phrases were combined into a word cloud with size of the text is proportional to the frequency with which that specific word or phrase was found in the articles [14,[40], [41], [42],[44], [45], [46], [47],^51^,^52^].

Only one study, by Chouliara and colleagues [40], sought to understand what QoL meant to older patients with cancer. They found that older patients wanted to maintain an average quality of life, meaning: enjoying life, no severe pain, minimal disruption to everyday life, and the ability to put aside cancer-related worry. Other studies explored areas of QoL and a summary of the important factors that older patients associated with QoL is shown in Fig. 3.

#### 3.3.4 Decision-making involvement

Eight studies investigated patient preferences for DM involvement [[40], [41], [42], [43],^48^,^49^,^51^,^52^]. Studies recruited patients with breast, colorectal, lung cancer, and hepatocellular carcinoma.

Three studies found that older patients relied heavily on expert advice, with decisions often led by HCPs [42,43,51]. In Jensen and colleagues' study, patients primarily relied on their HCP's recommendation, with 78% reporting that they felt there was no choice regarding treatment [43]. Passivity in the DM process was more likely to occur where patients had trust in HCPs opinion [42,51]. Andersen and colleagues [49] observed that demographic factors, such as age, were strong indicators of DM patterns, finding that older women felt less involved in the DM process compared to younger women. Conversely, Harder and colleagues found that most older patients with breast cancer (58.5%) favoured a collaborative decision made with their HCP [41]. Wörns and colleagues [52] also observed collaboration between HCPs and their patients in the DM process. Jorgensen and colleagues found a greater preference for shared DM amongst younger patients compared to older patients, although the difference between groups was not significant (*p* = 0.12) [51].

#### 3.3.5 Available decision support interventions

One study [48] developed and tested a decision support intervention. Wyld and colleagues [48] demonstrated that decision support interventions were able to enhance shared DM and increase patient knowledge of treatment options. Their tool led to a 20% absolute increase the number of patients answering “Yes” to the question, “I know enough about the options available to me.” It also led to a statistically significant increase in knowledge (*p* < 0.001). Furthermore, the study found that the decision aid shifted treatment choice towards preservation of QoL after providing patients with personalized survival estimates.

## 4 Discussion

This review reports evidence that treatment decisions made by older patients with cancer may differ from younger patients. It is likely that older patients are prioritizing QoL (including both illness and treatment burden) over length-of-life outcomes of treatment, although few studies presented age-stratified analyses in their findings. As such, older patients either express, or can be inferred to desire, information about QoL outcomes, and they use this in trading-off between survival, illness, and treatment burdens when making decisions.

Cancer treatment DM is influenced by a variety of factors, including the desire to maintain QoL during treatment [53]. This is also reflected in our previous work in breast cancer care, where patients favoured information focusing on the impact of treatment on independence and physical function [54,55]. In respect to quality and length-of-life preferences, studies reported that older patients were more likely to prioritize QoL, in keeping with the wider literature [56,57]. This inclination could be attributed to patients placing greater importance on treatment side-effects and the implications these may have for their QoL [40,42,58].

Several tools exist to identify preferences for preserving QoL in patients with advanced cancer, such as the Quality-adjusted time without symptoms or toxicity (Q-TWiST) [59] and Quality Quantity Questionnaire (QQQ) [60]. However, none of these are validated for use in patients with early cancer or those who already have a limited life expectancy due to advanced age or frailty. Overall, the review found scant evidence of decision support tools developed for use in older populations, despite growing evidence of their effectiveness in cancer treatment DM [61,62] and, in particular, helping patients to weigh-up the impact of treatment on their QoL [63].

While a handful of studies reported that older patients were more passive in treatment DM involvement, this finding is not consistent in the wider literature [64,65], with passivity usually disappearing in a decision aid-supported consultation. There is also evidence that patients are more likely to perceive their decision as a choice [66] and have a better understanding of treatment information [51] after using a decision aid. Many factors influence the extent to which older patients choose to engage in treatment DM [57], and while some older patients lean towards HCP expertise, this does not negate their desire to be involved in the treatment DM process.

Pitching the right level and content of information is clearly important, as this enables patients to process and understand their treatment options without feeling overwhelmed by an avalanche of information that may not have personal relevance. A key limitation of cancer provision is that it often focuses primarily on preparing patients for treatment and addressing short-term outcomes, without always considering the longer-term impacts of treatment on the person's social setting. While immediate concerns are important, such as treatment plans and side-effects, there is often a lack of guidance around the impact of treatment on QoL in the long-term. In addition, the impacts of well-established or novel cancer treatment options in older adults are frequently not reported in registration trials in oncology [67]. These gaps of knowledge may add substantial uncertainty in discussion with older patients and their caregivers, leaving them unprepared for future challenges in the aftermath of cancer treatments. Ways to produce information that resonates with older patients could include the use of decision support resources, complemented by geriatric assessment summaries [68] and extended consultation discussions [17].

### 4.1 Limitations

Our study has several limitations with respect to bias. Although a thorough database search was used to systematically identify relevant literature, there is a potential for reviewer and selection bias. Several papers relevant to the topic were excluded on the basis that participant demographics, such as age, were unreported. Given the lack of studies carried out primarily in older patients, we chose to allow those with a mean age of at least 65 if the recruitment criteria was inclusive of patients aged over 70, or if the papers included an older age comparison group. In a number of papers, older patients were not the majority population group, and this could be seen as a limitation in terms of generalising results. The studies that reported outcomes broadly for mixed early and late-stage cancer groups may introduce bias, as stage can significantly impact the DM process. For example, patients with late-stage cancer may prioritize treatments that enhance QoL, even if such treatments do not offer a cure. As the review includes several cancer types, it is possible that the priorities of older patients may differ depending on cancer type. For example, symptom burden on QoL, treatment options, and disease trajectory may differ between patients with breast and head and neck cancer. Despite the significant role of caregivers, particularly their involvement in the support system of older adults with cancer, none of the studies included caregivers or family members in their study populations.

### 4.2 Implications for policy and practice

The review highlights the need for a patient-centred approach to treatment DM that considers the heterogeneity of older adults. This reflects consensus from the American Society of Clinical Oncology (ASCO), the International Society of Geriatric Oncology (SIOG), and the National Comprehensive Cancer Network (NCCN), which recommend the use of geriatric assessments to inform treatment discussions with older adults with cancer [[69], [70], [71]]. The impact of treatment on QoL can be profound for all patients with cancer, both young and old, and this necessitates conversations that recognize the priorities and values of individuals as part of the DM discussion. This should include encouraging conversations that explicitly address the trade-offs between length and QoL. Research indicates that decision aids have the potential to enhance treatment DM in consultations with older patients with cancer [68]. Despite this, the uptake in decision aid use remains low in clinical practice.

### 4.3 Recommendations for future research

Future research should explore the intersectionality of socioeconomic factors that may influence the DM of older patients. This was highlighted in our EDI analysis, where few studies reported on the relevance of ethnicity and socio-economic status. Additionally, research should focus more on the ways in which baseline patient health, frailty, and comorbidity burden may impact patient priorities. Prioritizing personalized multidisciplinary care, which is aligned with patient preferences, should also be a key focus. Although decision aids have the potential to increase patient engagement, existing tools may not adequately address the needs and preferences of older patients due to their design being based on data and research in younger populations. Further research is needed to design and evaluate decision support tools that assess the decision preferences of older adults with cancer [15,42], and are based on data from studies that are inclusive of older participants.

Oncology clinical trials should increasingly focus on reporting treatment outcomes that matter to patients, such as effects on QoL, function, and treatment tolerability, in this specific age group [72]. Expanding the evidence based on these aspects may enable more informed discussions on the pros and cons of cancer treatment decisions between clinicians and older adults. Ultimately, further research into this area will help HCPs to guide patients who wish to prioritize their independence and QoL in the final phase of their life and enable more patient-centred care in the future.

## 5 Conclusion

Our findings show that the majority of older adults are willing to trade off some degree of survival benefits or disease control to preserve quality of life and highlight the importance of providing tailored information that addresses the preferences of older patients when making cancer treatment decisions. There are indications that older patients would benefit from information on both quality and length-of-life when making decisions about cancer treatments, particularly relating to treatment impacts on physical wellbeing, autonomy, and symptom burden. There is limited research around what quality of life means to older adults with early cancer and on the use of decision support tools in this setting. More research is needed to understand the priorities of older adults with early cancer when making treatment decisions that may impact on their quality of life. Decision support tools have the potential to assist in trade-off discussions, but few have been developed to balance trade-offs between quality and length-of-life for older adults in the early cancer setting.

## Funding

This study is funded by the NIHR Advanced Fellowship Programme (NIHR302481). The views expressed are those of the author(s) and not necessarily those of the NIHR or the Department of Health and Social Care.

## Author Contribution

Conceptualisation: JM, LW.

Data curation: CM, JM, JB, JM

Formal analysis: CM, JM.

Funding acquisitionLBackspace: JM

Investigation: JM, CM, JB

Methodology: JM, CM, LW, HB, AE

Project administration: CM, JM.

Resources: CM, JM.

Software: N/A

Supervision: JM, LW, NMLB, HB, AE

Validation: CM, JM,

Visualisation: CM, JM

Writing - original draft: CM, JM.

Writing - review & editing: CM, JM, LW, JB, NMLB, HB, AE.

All authors have read and approved the final manuscript.

## Declaration of Competing Interest

NMLB: Advisory board: Pfizer, Abbott, Sanofi, Astellas; Travel grants: Exact Sciences, Pfizer, Lilly, Novartis; Speaker fees: Pfizer, AbbVie, Roche, Sanofi, Novartis, Servier, Gilead, AstraZeneca, Lilly.

## Appendix A Supplementary data

Supplementary material 1 Image 1

Supplementary material 2 Image 2

Supplementary material 3 Image 3
