# Supplementary material for: Treatment decision-making and quality of life versus length of life preferences of older patients with early stage cancer: A systematic review
Source: J Geriatr Oncol. 2025 Nov;16(8):102773. doi: 10.1016/j.jgo.2025.102773 (PMC12621356; doi:10.1016/j.jgo.2025.102773)
Supplement: Supplementary file 1 — Supplementary material 1 [file mmc1.docx]

**Supplemental data 1: Database search strategy (Ovid MEDLINE**)

1 exp Neoplasms/

2 (cancer* or tumo?r* or neoplasm* or oncolog*).ti,ab,kf.

3 1 or 2

4 exp Aged/

5 (elder* or older patient* or older adult* or geriatric or old age* or frail*).ti,ab,kf.

6 4 or 5

7 exp "Quality of Life"/

8 (quality of life or QoL).ti,ab,kf.

9 7 or 8

10 Longevity/ or exp Life expectancy/

11 (length of life or LoL or longevity or longer life or survival or life expectancy).ti,ab,kf.

12 10 or 11

13 9 and 12

14 (quality vs length or quality versus length).ti,ab,kf.

15 Quality-Adjusted Life Years/

16 14 or 15

17 13 or 16

18 exp Decision Making/ or exp Decision Support Techniques/ or Patient Preference/ or Patient Participation/

19 (decision-making or decision tool* or decision support or decision aid* or trade-off or patient preference* or patient choice or treatment choice).ti,ab,kf.

20 18 or 19

21 3 and 6 and 17 and 20
