# Supplementary material for: Treatment decision-making and quality of life versus length of life preferences of older patients with early stage cancer: A systematic review
Source: J Geriatr Oncol. 2025 Nov;16(8):102773. doi: 10.1016/j.jgo.2025.102773 (PMC12621356; doi:10.1016/j.jgo.2025.102773)
Supplement: Supplementary file 2 — Supplementary material 2 [file mmc2.docx]

Supplemental Table 1: MMAT Quality appraisal of included studies

| Study ID | Study type | | | | | | |
| --- | --- | --- | --- | --- | --- | --- | --- |
|  | Qualitative studies | | | | | | |
|  | S1. Are there clear research questions? | S2. Do the collected data allow to address the research questions? | 1.1. Is the qualitative approach appropriate to answer the research question? | 1.2. Are the qualitative data collection methods adequate to address the research question? | 1.3. Are the findings adequately derived from the data? | 1.4. Is the interpretation of results sufficiently substantiated by data? | 1.5. Is there coherence between qualitative data sources, collection, analysis and interpretation? |
| **Chouliara et al. 2004** | Y | Y | Y | Y | Y | Y | Y |
| **Husain et al. 2007** | Y | Y | Y | Y | Y | Y | Y |
| **Wörns et al. 2024** | Y | Y | Y | Y | Y | Y | Y |
|  | Randomised controlled trials | | | | | | |
|  | S1. Are there clear research questions? | S2. Do the collected data allow to address the research questions? | 2.1. Is randomization appropriately performed? | 2.2. Are the groups comparable at baseline? | 2.3. Are there complete outcome data? | 2.4. Are outcome assessors blinded to the intervention provided? | 2.5 Did the participants adhere to the assigned intervention? |
| **Wyld et al. 2021** | Y | Y | Y | Y | Y | Y | Y |
|  | Quantitative descriptive studies | | | | | | |
|  | S1. Are there clear research questions? | S2. Do the collected data allow to address the research questions? | 4.1. Is the sampling strategy relevant to address the research question? | 4.2. Is the sample representative of the target population? | 4.3. Are the measurements appropriate? | 4.4. Is the risk of nonresponse bias low? | 4.5. Is the statistical analysis appropriate to answer the research question? |
| **van Tol-Geerdink et al. 2006** | Y | Y | Y | Y | Y | ? | Y |
| **Andersen et al. 1999** | Y | Y | Y | Y | Y | N | Y |
| **Watson et al. 2020** | Y | Y | Y | Y | Y | ? | Y |
| **Noordman et al. 2018** | Y | Y | Y | Y | Y | Y | Y |
| **Jansen et al. 2004** | Y | Y | Y | Y | Y | ? | Y |
| **Kool et al. 2016** | Y | Y | Y | N | Y | ? | Y |
| **Dhakal et al. 2022** | Y | Y | Y | ? | Y | ? | Y |
| **Jorgensen et al 2013** | Y | Y | Y | ? | Y | ? | Y |
| **Yellen and Cella 1994** | Y | Y | Y | ? | Y | ? | Y |
|  | Mixed methods studies | | | | | | |
|  | S1. Are there clear research questions? | S2. Do the collected data allow to address the research questions? | 5.1. Is there an adequate rationale for using a mixed methods design to address the research question? | 5.2. Are the different components of the study effectively integrated to answer the research question? | 5.3. Are the outputs of the integration of qualitative and quantitative components adequately interpreted? | 5.4. Are divergences and inconsistencies between quantitative and qualitative results adequately addressed? | 5.5. Do the different components of the study adhere to the quality criteria of each tradition of the methods involved? |
| **Harder et al 2013** | Y | Y | Y | Y | Y | ? | Y |

Y = Yes; N = No; ? = Can’t tell
