# Supplementary material for: Treatment decision-making and quality of life versus length of life preferences of older patients with early stage cancer: A systematic review
Source: J Geriatr Oncol. 2025 Nov;16(8):102773. doi: 10.1016/j.jgo.2025.102773 (PMC12621356; doi:10.1016/j.jgo.2025.102773)
Supplement: Supplementary file 3 — Supplementary material 3 [file mmc3.docx]

Supplemental Table 2: Equality, Diversity and Inclusivity Assessment of included papers

| **Study** | **Place of Residence** | **Race, ethnicity, language** | **Occupation** | **Gender/Sex** | **Religion** | **Education** | **Socio-economic status** | **Social Capital** | **Plus (age, disability, comorbidity, time)** | **Total score (out of 9)** |
| --- | --- | --- | --- | --- | --- | --- | --- | --- | --- | --- |
| **Andersen et al. 1999^(49)^** | 0 | 0.5 | 0 | 1 | 0 | 1 | 0.5 | 0 | 1 | **4** |
| **Chouliara et al. 2004^(40)^** | 0 | 0 | 0 | 1 | 0 | 0 | 0 | 0 | 1 | **2** |
| **Dhakal et al. 2022^(14)^** | 0 | 0.5 | 0 | 1 | 0 | 0.5 | 0.5 | 0 | 1 | **3.5** |
| **Harder et al. 2013^(41)^** | 0 | 0 | 0 | 1 | 0 | 0 | 0 | 0 | 1 | **2** |
| **Husain et al. 2007^(42)^** | 0 | 0 | 0 | 1 | 0 | 0 | 0 | 0 | 1 | **2** |
| **Jansen et al. 2004^(43)^** | 0 | 0 | 0 | 1 | 0 | 1 | 0.5 | 0 | 1 | **3.5** |
| **Jorgensen et al. 2013^(51)^** | 0 | 0 | 0 | 1 | 0 | 0.5 | 0.5 | 0 | 1 | **3** |
| **Kool et al. 2016^(44)^** | 0 | 0 | 0 | 1 | 0 | 1 | 0.5 | 0 | 1 | **3.5** |
| **Noordman et al. 2018 ^(45)^** | 0 | 0 | 0 | 1 | 0 | 0.5 | 0.5 | 0 | 1 | **3** |
| **van Tol-Geerdink et al. 2006 ^(46)^** | 0 | 0 | 0 | 1 | 0 | 0.5 | 0.5 | 0 | 1 | **3** |
| **Watson et al. 2020^(47)^** | 0 | 0 | 0 | 1 | 0 | 0.5 | 0.5 | 0 | 1 | **3** |
| **Wörns et al. 2024^(52)^** | 0.5 | 0.5 | 0 | 1 | 0 | 0.5 | 0 | 0.5 | 1 | **4** |
| **Wyld et al. 2021^(48)^** | 0 | 0 | 0 | 1 | 0 | 0.5 | 0.5 | 0 | 1 | **3** |
| **Yellen and Cella. 1994^(50)^** | 0 | 0 | 0 | 1 | 0 | 0.5 | 0.5 | 0 | 1 | **3** |

*0= not reported in the paper; 0.5= partially reported (ie. collected but not analysed); 1= analysed/reported/measured
